# Foliose *Ulva* Species Show Considerable Inter‐Specific Genetic Diversity, Low Intra‐Specific Genetic Variation, and the Rare Occurrence of Inter‐Specific Hybrids in the Wild

**DOI:** 10.1111/jpy.13079

**Published:** 2020-11-24

**Authors:** Antoine Fort, Marcus McHale, Kevin Cascella, Philippe Potin, Björn Usadel, Michael D. Guiry, Ronan Sulpice

**Affiliations:** ^1^ Plant Systems Biology Lab Ryan Institute & MaREI Centre for Marine Climate and Energy School of Natural Sciences National University of Ireland ‐ Galway Galway H91 TK33 Ireland; ^2^ UMR 8227 Integrative Biology of Marine Models CNRS Sorbonne Université Sciences Station Biologique de Roscoff, CS 90074 F‐29688 Roscoff France; ^3^ Institute for Biology I RWTH Aachen University Worringer Weg 3 Aachen 52074 Germany; ^4^ AlgaeBase Ryan Institute National University of Ireland Galway H91 TK33 Ireland

**Keywords:** macroalgae, next‐generation sequencing, phylogeny, sexual reproduction, *Ulva*

## Abstract

Foliose *Ulva* spp. have become increasingly important worldwide for their environmental and financial impacts. A large number of such *Ulva* species have rapid reproduction and proliferation habits, which explains why they are responsible for *Ulva* blooms, known as “green tides”, having dramatic negative effects on coastal ecosystems, but also making them attractive for aquaculture applications. Despite the increasing interest in the genus *Ulva*, particularly on the larger foliose species for aquaculture, their inter‐ and intra‐specific genetic diversity is still poorly described. We compared the cytoplasmic genome (chloroplast and mitochondrion) of 110 strains of large distromatic foliose *Ulva* from Ireland, Brittany (France), the Netherlands and Portugal. We found six different species, with high levels of inter‐specific genetic diversity, despite highly similar or overlapping morphologies. Genetic variation was as high as 82 SNPs/kb between *Ulva pseudorotundata* and *U*. *laetevirens*, indicating considerable genetic diversity. On the other hand, intra‐specific genetic diversity was relatively low, with only 36 variant sites (0.03 SNPs/kb) in the mitochondrial genome of the 29 *Ulva rigida* individuals found in this study, despite different geographical origins. The use of next‐generation sequencing allowed for the detection of a single inter‐species hybrid between two genetically closely related species, *U*. *laetevirens*, and *U*. *rigida*, among the 110 strains analyzed in this study. Altogether, this study represents an important advance in our understanding of *Ulva* biology and provides genetic information for genomic selection of large foliose strains in aquaculture.

**Abbreviations**
*tuf*A: elongation factor Tu
SNP: Single Nucleotide Polymorphism
MCMC: Markov Chain Monte Carlo
GMYC: Generalized Mixed Yule Coalescent
NCBI: National Center for Biotechnology Information

The genus *Ulva* (Ulvaceae, Ulvophyceae) encompasses a large group of green macroalgae species found throughout the world’s oceans, with some 132 distromatic foliose and generally monostromatic tubular species (*Enteromorpha*‐like) currently accepted taxonomically, and several species displaying both morphotypes (Guiry and Guiry 2020). Distromatic foliose *Ulva* species show two‐cell thick sheet‐like thalli (Hofmann et al. 2010), giving the genus its vernacular name of “Sea Lettuce”. Monostromatic tubular species, which were previously thought to belong to a distinct genera (*Enteromorpha* spp.; Hayden et al. 2003), typically form tubular thalli that are one cell thick. Species of the genus have considerable environmental impact due to their ability to generate “green tides,” a phenomenon whereby extensive mats of biomass cover large coastal areas, with strong negative impacts for marine intertidal and subtidal ecosystems and tourism industries (Yabe et al. 2009, Ye et al. 2011, Wang et al. 2012, Gao et al. 2014). Both morphotypes have been shown to be able to generate green tides in Europe and Asia (Coat et al. 1998, Taylor et al. 2001, Hiraoka et al. 2004, Zhang et al. 2013a, Wan et al. 2017, Fort et al. 2020). By contrast, *Ulva* has considerable economic potential as it is grown in aquaculture as food and feed, either in specialized production systems (Ghaderiardakani et al. 2019, Magnusson et al. 2019) or in integrated multi‐trophic aquaculture (Marinho et al. 2013, Amosu et al. 2016). Foliose *Ulva* species are also recently being exploited for production of bioactive compounds, such as ulvans (Adrien et al. 2019, Reis et al. 2020). With increasing production, the domestication of *Ulva* spp. now presents exciting opportunities. High levels of genetic variation among foliose *Ulva* strains are associated with large differences in biomass production (Fort et al. 2019, 2020). Such genetic variation presents foliose *Ulva* as an attractive target for genomic improvement. The first step toward such improvement is the characterization of this diversity and its relationship to industrial, economic, and environmental properties of foliose *Ulva* spp. (Fort et al. 2020). The complete genome of a single *Ulva* species (*Ulva mutabilis*, now considered conspecific with *Ulva compressa*; Steinhagen et al. 2019a) provides a useful reference for future genomic work (De Clerck et al. 2018). To date, however, the extent of genetic diversity among large foliose *Ulva* spp. has not been investigated in detail other than few genic regions sequenced as “barcodes” (O’Kelly et al. 2010, Kirkendale et al. 2013, Miladi et al. 2018). Also, only nine mitochondrial and eight chloroplast genomes of *Ulva* species have been sequenced fully, and the degree of cytoplasmic genetic variation among strains of the same species is unknown. Furthermore, typical barcoding of cytoplasmic markers or even full cytoplasmic sequencing does not allow for the detection of inter‐specific hybrids. The rapidly decreasing cost of high‐throughput sequencing combined with a cheap and efficient DNA extraction method (Fort et al. 2018) has allowed us to conduct a genetic study of the foliose *Ulva* species complex in the North East Atlantic.

We analyzed the cytoplasmic genome and a repetitive genomic marker (45S) of 110 foliose *Ulva* strains sampled at various locations in Ireland, Brittany (France), the Netherlands and Portugal. This dataset allowed us to i) characterize the extent of inter‐ and intra‐specific cytoplasmic genetic variation among those foliose *Ulva* species and ii) question the possibility of hybridization between *Ulva* individuals from different foliose species in the wild.

## MATERIALS AND METHODS

### DNA extraction and sequencing

Individual thalli of intertidal and subtidal distromatic foliose *Ulva* individuals were collected between January and October 2018 at different locations in Europe, representing diverse habitats (rocky shores, estuaries, beaches). Only *Ulva* samples with a foliose thallus larger than ~ 100 cm^2^ were kept for further analysis. Samples were immediately placed in clip‐seal bags filled with chilled seawater and transported to the laboratories in insulated boxes. For a list of samples and their origin see Table S1 in the Supporting Information. The *Ulva* thalli were sent to Ireland in a similar manner and upon arrival an ~ 4‐5 cm in diameter piece of each thallus was cut, snap frozen in liquid nitrogen and stored at −80°C. Samples were then freeze‐dried and ground into a fine powder using a ball mill (QIAGEN Tissue Lyser II). DNA was extracted from ~ 7‐8 mg dry weight of each sample using magnetic beads (Fort et al. 2018). The DNA from 110 samples was submitted to Illumina 150 bp paired‐end sequencing (Novogene Ltd, Hong Kong). At least 3 Gbp of data was obtained from each sample.

In parallel, DNA was extracted from ~ 4 g of tissue from an Irish strain (U41, Courtmacsherry, County Cork) and subjected to sequencing using Oxford Nanopore MinION technology (Schmidt et al. 2017).

### Species delimitation and phylogenetic relationship reconstruction

**Fig. 1:**
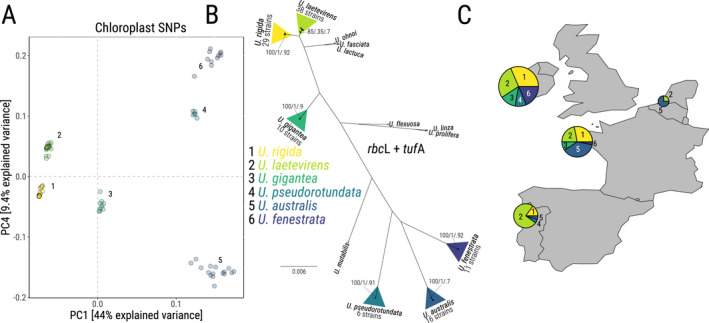
Samples analyzed in this study. (A) Clustering of strains based on their chloroplast genome. Principal Component Analysis (PC1 and PC5) on SNP data for each strain against the chloroplast genome of strain U41’s nanopore assembly. Full PCA available in Figure S2. Shading represents the six clusters found. (B) Phylogram of the 110 sampled individuals based on *rbc*L and *tuf*A concatenated sequences based on MCMC Bayesian analysis. Numbers represent support for clade clusters (first number: Bayesian posterior probability of clades, second number: GMYC support, third number: Bayesian Poisson Tree Processes support). Triangles represent the assigned species to the cluster. Scale bar = expected changes per site. (C) Sampling locations and relative abundance of each foliose *Ulva* species. Numbers represent the six clusters. Full list and detailed coordinates available in Table S1.

For all phylogenetic analysis, sequences were aligned using MUSCLE (Edgar 2004). For species delimitation (Fig. 1B), Ribulose biphosphate carboxylase large chain (*rbc*L) and elongation factor Tu (*tuf*A) sequences from the 110 strains were recovered from the chloroplast genome (see Organellar genome analysis section), as well as the *rbc*L and *tuf*A sequences recovered from seven previously published *Ulva* chloroplast sequences. We used two distinct methods to determine species delimitation. First, the alignments were analyzed under a General Mixed Yule Coalescent (GMYC) model (Pons et al. 2006) in BEAST (Bouckaert et al. 2014) using a General Time Reversible (GTR) nucleotide substitution model (Arenas 2015), including 4 Gamma categories (G) and a proportion of invariant sites (I), a strict clock and 1 million Markov Chain Monte Carlo (MCMC). The most adequate substitution parameters for the phylogenetic analyses were determined using jModelTest, and GTR + G + I was found to be the most appropriate based on Akaike and Bayesian information criteria (AIC and BIC, respectively; Darriba et al. 2012). Tracer (Rambaut et al. 2015, 2018) was used to analyze the posterior estimates of the BEAST run, and confirmed convergence of the chain and adequate Estimated Sample Size (ESS) score> 200. The trees obtained were summarized using TreeAnnotator (Rambaut and Drummond 2015, 2018), and species delimitation was performed on the highest scoring tree using the rncl and splits packages in R (Fujisawa and Barraclough 2013). Second, the alignments were analyzed with MrBayes (same GTR + G + I model) to obtain the posterior probability of clades and average branch lengths (Average standard deviation of split frequencies after 1,000,000 generations: 0.0045, indicating chain convergence; Huelsenbeck and Ronquist 2001). The consensus three obtained from the Bayesian phylogenetic analysis was analyzed using the Bayesian Poisson Tree Process (bPTP) for species delimitation (Zhang et al. 2013b) with 1,000,000 MCMC generations. Analysis of the log likelihood indicated convergence (Fig. S1 in the Supporting Information). For maximum likelihood phylogenetic trees, alignments were analyzed using RaxML (Stamatakis 2014). Statistical support was estimated using 1,000 bootstraps. Sequences from published mitochondrial or chloroplast *Ulva* genomes were included in the tree. Tree figures were produced with the Figtree software (http://tree.bio.ed.ac.uk/software/figtree/) and edited in Inkscape (https://inkscape.org/).

### Organellar genome assemblies

The Nanopore reads of strain U41 were assembled using Canu (Koren et al. 2017) to generate long contigs from an *Ulva* holobiont. The long read assembly was then polished with U41 Illumina sequences using pilon (Walker et al. 2014). Contigs flagged as circular from this polished assembly were compared to the National Center for Biotechnology Information (NCBI) nucleotide database. For both mitochondrial and chloroplast genomes a single circular contig was identified with high BLAST scores to the relevant published Ulva sequences.

We initially mapped all the reads from the 110 strains to the chloroplast contig of the nanopore assembly to identify possible species clusters within the dataset. Reads were mapped using bowtie2 (Langmead and Salzberg 2012), and variant calling performed using BCFtools (Li 2011). The resulting variant files were analyzed in R using the package SNPRelate to perform Principal Component Analysis (Zheng et al. 2012). The clustering of strains based on their chloroplast SNP data revealed the presence of six species clusters within the dataset (Figs. 1A and S2 in the Supporting Information).

Then, we developed a multi‐step process of filtering and assembly to produce reference organellar genomes for each of the six species present. Contigs and available chloroplast or mitochondrial genomes of *Ulva* species were used as starting points for de novo organelle assembly using SPADes (Bankevich et al. 2012). This was performed independently for each of the 6 species present in our dataset and separately for each cytoplasmic genome, either mitochondrial or chloroplast. We used an iterative approach designed to i) address significant sequence variation between each species and available references and ii) mitigate the hardware requirements for such analysis (Fig. S3 in the Supporting Information): first, raw reads from one strain per species were filtered based on mapping to either our U41 reference contig or available published genomes of the relevant organelle type. These organelle‐specific reads were input to SPADEs for de novo genome assembly. In most cases (apart from *U*. *laetevirens*, which is the species of strain U41), we failed to create a unique circular contig in this first iteration of de novo assembly. This result indicates that most species contain sequences not present in the available reference genomes. As such, for all other species, the scaffolds obtained from SPADes were used to again filter the Illumina reads and perform de novo assembly from this filtered set. This procedure was repeated until a single circular contig was obtained (Fig. S3). Circularity of the contig at each iteration was assessed using Bandage (Wick et al. 2015). Each iteration allows the “overhangs” of reads at the junction loci to be added to the new assembly, filling the gaps of species‐specific genomic regions that are not represented in the other sequenced *Ulva* species organelles, while limiting the usage of memory due to a pre‐filtering step of reads.

### Organellar genome analysis

Illumina sequences from all strains were mapped against their species‐specific reference organellar genome. A consensus sequence was then constructed for each strain using variant calls from bcftools mpileup (Li 2011). Each strain’s organellar reference was annotated using GeSeq (Figs. S4 and S5 in the Supporting Information; Tillich et al. 2017) and gene coordinates for each strain used to extract the coding sequences from a common set of annotated genes (CDS; 69 and 29 genes for chloroplast and mitochondrion, respectively; Table S2 in the Supporting Information). These CDS were concatenated for phylogenetic analysis as described in “Species delimitation and phylogenetic relationship reconstruction”.

The number of Single Nucleotide Polymorphisms (SNPs) per strain within these sequences was also determined relative to the consensus for *Ulva laetevirens*. We chose *U*. *laetevirens* as reference here due to its high presence across the sampled sites (Fig. 1C). The mean SNPs per species against *U*. *laetevirens* was then calculated and normalized by length. The extent of intra‐specific variation was assessed by considering the mean incidence of SNPs per strain relative to the consensus organellar genome for the given species and normalizing this count by length.

For synteny analysis, we used progressive Mauve (Darling et al. 2010) to identify syntenic blocs between *Ulva* species. Visualization of the detected inversion between *U*. *rigida*, *U*. *laetevirens*, *U*. *gigantea* and *U*. *pseudorotundata [≡ U. rotundata]*, *U*. *fenestrata*, *U*. *australis*, as well as the SNP density in *U*. *fenestrata* chloroplast genome were developed using Circos (Krzywinski et al. 2009).

Principal Component Analyses (PCA) were performed using the R package SNPRelate (Zheng et al. 2012) on the SNPs in each strain’s mitochondrial or chloroplast genomes.

### 45S repeats sequence identification

The ribosomal RNA 45S repeats were readily identified by mapping Illumina reads against the Nanopore assembly due to their high coverage (>1000x) and homology of this mapped region to *Ulva* rRNA sequences published on NCBI (*Ulva*. *expansa* MH730160, MH730161, and *U*. *prolifera* KY350852, respectively). The Illumina reads from the 110 strains were then mapped onto this locus in the Nanopore assembly and variant calling was performed as described in “Organellar Genome Analysis.”

## RESULTS

### High‐throughput sequencing

In order precisely to characterize the organellar genomic diversity of wild foliose *Ulva* spp. in the North East Atlantic, we employed a next‐generation sequencing approach. We sampled 110 foliose *Ulva* individuals from 4 countries in Europe at 23 different locations, with 1‐5 *Ulva* individuals per site (Fig. 1; for the list of samples and their precise geographical origin, see Table S1). Each individual was sequenced using the Illumina platform, and a single individual (U41) was sequenced with both Illumina and Oxford Nanopore MinION long reads technologies, to serve as a reference anchor to facilitate organellar genome assembly. We obtained > 10 M paired‐end reads for each sample, containing nuclear, organellar, and bacterial reads. The abundance of bacterial reads was significant and not unexpected, due to the described symbiotic relationship between *Ulva* and several bacterial species (Marshall et al. 2006, Spoerner et al. 2012). Thus, given this limitation, we focused our analysis on organellar genomes and the 45S ribosomal RNA marker due to their high copy number sequences.

### Reconstruction of organellar genomes of foliose *Ulva* spp

To determine the number of species and support their identification within the 110 strains in our dataset, we first analyzed the clustering of strains based on SNPs present within their chloroplast genome by PCA. This analysis revealed the presence of 6 distinct clusters within the dataset (Fig. 1A and Fig S2). For species identification, we extracted the reads mapping onto the two chloroplast barcodes traditionally used for species identification in *Ulva*, *rbc*L, and *tuf*A (Kirkendale et al. 2013, Du et al. 2014, Lee et al. 2019). The NCBI reference for each barcode in each strain is available in Table S1. Based on the sequence of both genes, we reconstructed a phylogenetic tree using Bayesian MCMC analysis (Arvestad et al. 2003), with species delimitation assessed with a Generalized Mixed Yule Coalescent model and a Bayesian Poisson Test Process (Esselstyn et al. 2012, Zhang et al. 2013b, Luo et al. 2018). The analysis confirmed that our dataset contains 6 distinct clusters, indicating the presence of six different *Ulva* taxa. Five of the six species were easily identified (*Ulva gigantea*, *Ulva pseudorotundata*, *Ulva australis*, *Ulva fenestrata,* and *Ulva rigida)* due to their large inter‐specific genetic variation (support for speciation = 1 under the Yule model, and 0.9, 0.91, 0.7, 0.92, and 0.92 under the Poisson test, respectively; Fig. 1B). Species assignment was based on identity with published sequences (*U*. *gigantea*: 100% identity with voucher HQ610297, *U*. *pseudorotundata*: 100% identity with voucher EU484406, *U*. *australis*: 100% identity with voucher LC507117, *U*. *fenestrata*: 100% identity with voucher MK456393, *U*. *rigida*: 100% identity with voucher AY422564). The last cluster was identified as monophyletic with the Poisson Test (support of 0.7), but only weakly supported using the Yule model (support of 0.35). This indicates that the cluster is likely monophyletic but may contain sub‐groups. However, the sub‐division appears to be caused by the presence of a single shared (SNP) in four strains compared with the other 34 strains of this cluster, indicating that all individuals from this cluster likely belong to the same species. The strains within that cluster were assigned to *Ulva laetevirens* based on the 100% sequence identity with the *tuf*A sequence of the type locality of *U*. *laetevirens* (NCBI accession number JN029327; Kirkendale et al. 2013).

**Fig. 2:**
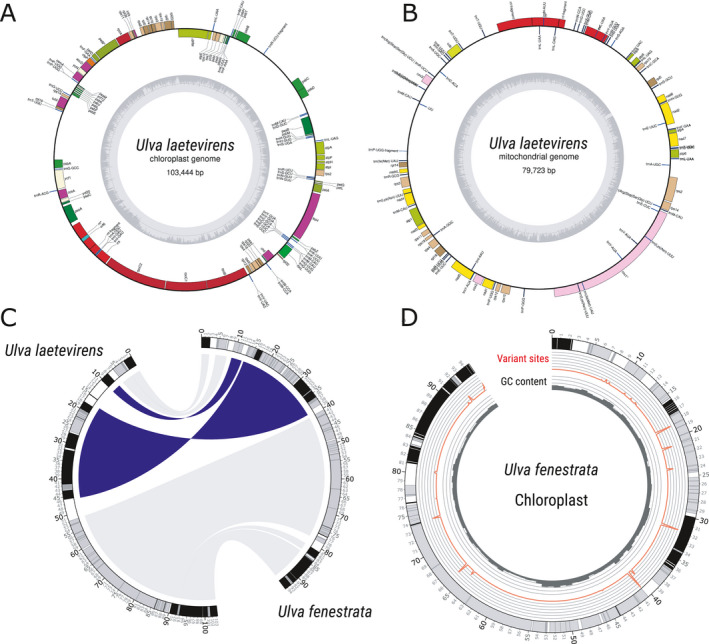
*Ulva* organelles assembly and annotation. (A) Annotation of *U. laetevirens* chloroplast. B) Annotation of *U. laetevirens* mitochondrion. The annotation of the other species is available in Figures S3 and S4. (C) Detected inversion between *U. laetevirens* and *U. fenestrata* chloroplasts. Inversion is shaded in dark, syntenic blocs in the same orientation are shaded in gray. Ideogram represent coding sequence (genes), black shading indicates plus strand, gray shading the minus strand. Numbers indicate positions in kilobases (D) Variant sites (SNPs) detected within the chloroplast of the 11 *U. fenestrata* samples. Line represents variant sites, and their relative abundance between strains. Histogram represents GC content, ideogram same as (A).

With a species determined for each strain in the dataset, we used a custom pipeline to assemble complete organellar genomes (mitochondrion and chloroplast, see “Organellar genome assemblies”) for a representative strain of the six species used in this study (Figs. 2, A and B; S4, NCBI references for each of the six species organelle genomes available in Table S1). We detected significant variation in the chloroplast genome size among the 6 species (95 kb to 118 kb; Table 1). The number of predicted genes was very stable with 101 to 102 across the sampled species, indicating that genome size differences are likely due to variation in the length of non‐coding regions. Interestingly, synteny analysis revealed the presence of a large inversion between *Ulva rigida*/ *U*. *laetevirens*/ *U*. *gigantea* and *U*. *australis*/ *U*. *pseudorotundata*/ *U*. *fenestrata* (Fig. 2C, Fig. S6 in the Supporting Information).

**Table 1:** Summary of the predicted annotation of the chloroplast genome of the 6 species found in this study.

Species	Strain	Size [bp]	Number of genes	tRNAs	Protein‐coding	Ribosomal RNA
*U. australis*	U100	99,820	102	26	74	2
*U. fenestrata*	U64	94,654	100	26	72	2
*U. pseudorotundata*	U112	118,206	102	28	72	2
*U. gigantea*	U57	117,606	101	27	72	2
*U. rigida*	U36	96,673	101	28	71	2
*U. laetevirens*	U35	103,444	101	27	72	2

The mitochondrial genome sizes ranged from 59 to 89 kb and contained 57 to 61 genes (Table 2; Fig. S5). We did not find any syntenic difference between the six species in our dataset and the previously published *Ulva* mitochondrial genomes (Fig. S7 in the Supporting Information).

**Table 2:** Summary of the predicted annotation of the mitochondrial genome of the 6 species found in this study.

Species	Strain	Size [bp]	Number of genes	tRNAs	Protein‐coding	Ribosomal RNA
*U. australis*	U100	64,466	57	26	29	2
*U. fenestrata*	U64	59,026	59	28	29	2
*U. pseudorotundata*	U112	88,416	59	28	29	2
*U. gigantea*	U57	66,743	58	27	29	2
*U. rigida*	U36	88,318	61	30	29	2
*U. laetevirens*	U35	79,723	61	30	29	2

In the chloroplast, strong genetic differences were identified between the 6 species, with for example ca. 90 SNPs per kb of coding sequence between *Ulva pseudorotundata* and *U*. *laetevirens* (Table 3), but intra‐specific variation was an order of magnitude lower. For instance, as little as 30 SNPs were identified between the 11 *U*. *fenestrata* individuals (0.12 SNPs · kb^‐1^ on average), despite originating from widely different geographical locations (Table 4; Fig. 2D).We observed the same pattern for the inter‐ and intra‐specific mitochondrial genetic variation (Tables 3 and 4) as for the chloroplast, with high levels of variation found between species, and low levels found within species. However, the mitochondrial inter‐specific genetic variation was generally higher than that seen for the chloroplast, with as many as 222 SNPs · kb^‐1^ of coding sequence between *U*. *pseudorotundata* and *U*. *laetevirens* (Table 3). Low level variation within species was also observed in mitochondria, with variation ranging from 1.36 to 0.03 SNPs · kb^‐1^. Only 14 variant sites were found between the 11 *U*. *fenestrata* individuals (Table 4).

**Table 3:** Between species genetic variation in *Ulva* cytoplasmic coding sequence. Numbers represent the average number of SNPs per kilobase, and number in brackets represent the average number of SNPs in the entire coding sequence between strains of each species and *U. laetevirens* strain U35.

Species	Chloroplast	Mitochondria
	Number of SNPs · kb^‐1^ Versus *U. laetevirens* (CDS)	Number of SNPs · kb^‐1^ Versus *U. laetevirens* (CDS)
*U. australis*	82 (5,230)	196 (4,860)
*U. fenestrata*	79.8 (5,090)	194 (4,826)
*U. pseudorotundata*	91.5 (5,836)	222 (5,512)
*U. gigantea*	35.3 (2,249)	86.1 (2,139)
*U. rigida*	6.1 (391)	5.6 (140)
*U. laetevirens*	0.6 (36)	0.03 (9)

**Table 4:** Within species genetic variation in *Ulva* cytoplasmic genome. SNPs/kb represents the average number of SNPs in 1000 bp in each strain ± SD. Total variant sites represent the total amount of SNPs identified within strains of the same species.

Species	Strains	Chloroplast		Mitochondria	
		SNPs · kb^‐1^	Total variant sites within species	SNPs · kb^‐1^	Total variant sites within species
*U. australis*	16	1.98 ± 0.66	392	1.05 ± 0.9	190
*U. fenestrata*	11	0.12 ± 0.07	30	0.05 ± 0.05	14
*U. pseudorotundata*	6	0.09 ± 0.09	44	0.04 ± 0.4	13
*U. gigantea*	10	0.38 ± 0.15	126	0.26 ± 0.09	43
*U. rigida*	29	0.18 ± 0.14	117	0.03 ± 0.05	36
*U. laetevirens*	38	1.14 ± 0.7	759	1.36 ± 0.7	578

### Comparison of species delimitation by use of barcoding and full organelle coding sequences

**Fig. 3:**
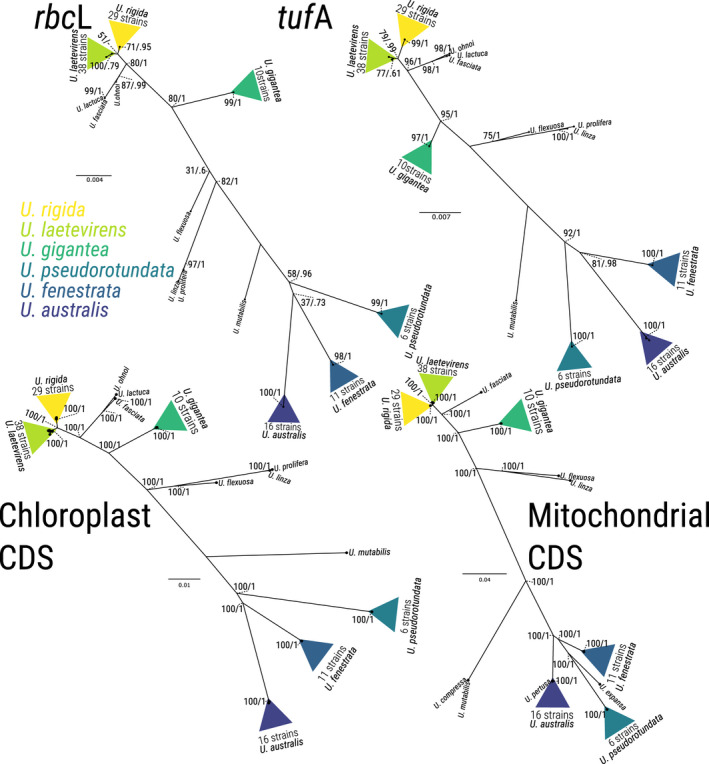
Agreement between barcoding and organelle coding sequence phylogenetic trees. Maximum Likelihood trees were constructed from the sequences of *rbc*L, *tuf*A, and the entire coding sequence of the organelles. Triangles indicate species clusters, numbers indicate the bootstrap support (left) and Bayesian posterior probability (right) for the major nodes of the tree.

Phylogenetic reconstruction from barcode sequences depends upon consistent variability in the regions considered for the analysis. In such reports for *Ulva* spp., the cytoplasmic *rbc*L and *tuf*A are widely used, with, although less frequently, the nuclear ITS sequences from the 45S ribosomal RNA, as well as 5S rDNA also being considered (Coat et al. 1998, Zhang et al. 2014). We aimed to assess whether these traditionally used barcodes are appropriate for *Ulva* species delimitation. We generated maximum likelihood phylogenetic trees for the entire set of coding sequence of the organellar genomes (63,775 bp and 24,854 bp for chloroplast and mitochondria, respectively), and compared the trees with those obtained for *rbc*L and *tuf*A. We found a complete agreement in the clustering of strains between the different trees (Fig. 3). Indeed, all four trees show the same clusters representing all 6 species in the dataset, demonstrating that barcodes are largely sufficient for species identification in *Ulva*, with similar inferences obtained from either short barcode sequences or the larger organelle coding sequences.

### Agreement between organellar and nuclear markers reveals the rarity of hybridization events between foliose *Ulva* species

**Fig. 4:**
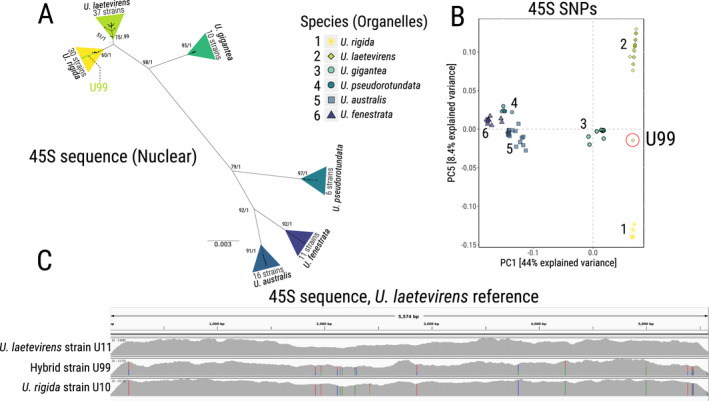
Detection of a single hybrid among the 110 foliose *Ulva* strains. (A) Maximum Likelihood phylogenetic tree based on the 45S nuclear sequence. Triangles indicate the inferred species of the strains from the organellar (cytoplasmic) data. Numbers indicate the bootstrap support for the major nodes (left) and Bayesian posterior probability (right). (B) Principal Component Analysis of SNPs detected in the 45S sequence. Strain U99 is circled. Numbers represent species clusters. (C) Coverage visualization of illumina reads on the 45S locus between a single strain of *U. laetevirens* and *U. rigida*, as well as the hybrid strain U99. Bars represent SNPs between the two species and their relative ratios.

*Ulva* sporophytes contain a single chloroplast genome per cell in vegetative thalli (Bråten 1973, Kagami et al. 2008) that is derived from either parent, while the nuclear diploid genome contains an equivalent mix of sequences derived from both parents. As a result, an *Ulva* inter‐specific hybrid is expected to contain a cytoplasmic (organellar) genome consistent with one parental species and a nuclear genome representing both parental species. To investigate whether *Ulva* species naturally hybridize in the wild, we mapped the Illumina reads against the nuclear‐encoded 45S ribosomal RNA repeats originating from our nanopore genomic assembly. We then generated a Maximum Likelihood phylogenetic tree using the nuclear 45S sequence for all strains and obtained a tree with striking similarity to that of either organellar genomes (Figs. 3 and 4A). In all but one case, strains were assigned into the same clusters using the 45S nuclear sequence and the organellar genomes. The single discrepancy found (strain U99) clustered with *U*. *rigida* in the analysis of nuclear 45S sequence and with *U*. *laetevirens* in analysis of organellar sequences. This result could indicate the presence of a hybrid, containing the nuclear genome of *U*. *rigida* and the cytoplasmic genome of *U*. *laetevirens*. Alternatively, this strain could represent an hybrid constituted of one copy of *U*. *rigida* genome and another of *U*. *laetevirens,* as well as the cytoplasmic genome of *U*. *laetevirens*. Indeed, the phylogenetic tree was constructed from each strain’s variant call consensus (VCF tools consensus) where a heterozygote is reduced to an annotation of presence of the minor allele, without further detail about potential heterozygosity. To consider more thoroughly any potential hybridization events, we performed a PCA on the minor/major allelic frequency. This analysis revealed only strain U99 as an outlier from relevant phylogenetic clusters (Fig. S8 in the Supporting Information). Importantly, this strain is positioned equidistant to both clusters in the discriminatory axis (PC5, Fig. 4B). Examination of the allelic frequencies in detail demonstrates that U99 is indeed heterozygous for all informative loci and is as such likely to be an F1 hybrid (Table S2). Figure 4C shows the Illumina read depth and highlighted bars correspond to informative SNP loci in the discrimination of strains U11 (*U*. *laetevirens*), U99 (hybrid), and U10 (*U*. *rigida*) with a color corresponding to the frequency of major or minor allele.

## DISCUSSION

### Foliose *Ulva* species show a large degree of cytoplasmic genetic variation

Phylogenetic analysis at the species level often relies on organellar sequences (Heesch et al. 2009, Miladi et al. 2018, Ruihua et al. 2018). Here, we used next‐generation sequencing and a custom analysis pipeline to reconstruct the organellar genomes of species of foliose *Ulva* spp. present along the European Atlantic coast. Our sampling strategy was intentionally blind to species identification, to allow for a comparison of the respective relative abundance of foliose *Ulva* species. The foliose morphotype of *Ulva* species was selected since foliose species are commonly cultivated in aquaculture and IMTA systems based on vegetative propagation methods (Bolton et al. 2009, Lawton et al. 2013, Shpigel et al. 2017, Califano et al. 2020). We detected the presence of six foliose species among the 110 sampled individuals: *Ulva rigida*, *U*. *laetevirens*, *U*. *gigantea*, *U*. *pseudorotundata*, *U*. *fenestrata,* and *U*. *australis*. Of those six species, *U*. *rigida* and *U*. *laetevirens* were the dominant species, particularly in Portugal, where 18 out of 22 samples were attributed to either of these taxa. The west Irish coast shows a similar species distribution, with 32 out of 55 individuals determined as *U*. *rigida* or *U*. *laetevirens*. By contrast, most individuals in Brittany (France) and the Netherlands were *U*. *australis*. Our findings are consistent with those of our previous study, involving similar locations in Europe using traditional PCR‐based barcoding (Fort et al. 2020). We did not find any *U*. *lactuca* individuals, in seeming contradiction to previous reports (Loughnane et al. 2008). However, a recent re‐examination of the nomenclatural types of *U*. *lactuca* and *U*. *fenestrata* has shown the former to be predominantly a southern hemisphere species, with reports of specimens from the European Atlantic of *U*. *lactuca* now being referred to *U*. *fenestrata* (Hughey et al. 2019). Our results confirm Hughey et al’s (2019) conclusions as we determined several individuals in Ireland, France, and Portugal as *U*. *fenestrata*. We also found the same range of large foliose species as described previously in the European coast when taking into account synonymous species names, with the exception of *Umbraulva* spp. (Tan et al. 1999, Loughnane et al. 2008, Steinhagen et al. 2019b), which in contrast to *Ulva* spp. are rare or uncommon throughout the NE Atlantic area (M. D. Guiry, pers. obs.).

However, the relative distribution of all species should be treated with caution as seasonality and over‐representation of some species in certain areas can amplify abundance results. Furthermore, seasonal variation between foliose *Ulva* species at the same localities has not been investigated, and further sampling at different timepoints and locations may reveal the presence of other species. Finally, the presence of *Ulva* species of foliose morphotype with small thalli (<100 cm^2^) could not be investigated here since we focused on large individuals.

While our sampling strategy is not necessarily expected to capture the entire foliose species diversity of the European Atlantic coast, the six species found here likely represent the most common large foliose *Ulva* species present in the Ireland, Portugal, Brittany, and the Netherlands. Indeed, the other main foliose *Ulva* species have to date not been found in the North East Atlantic: *Ulva lactuca* sensu stricto is a southern hemisphere/ warm waters species (Hughey et al. 2019),*Ulva expansa* is currently restricted to the Pacific (Hayden and Waaland 2004), *Ulva ohnoi* is present in warm waters (Mediterranean, where it is probably adventive, the Gulf of Mexico, Asia, Oceania; Hiraoka et al. 2004, Melton and Lopez‐Bautista 2016, Krupnik et al. 2018, Miladi et al. 2018), and *Ulva araskii* appears to be restricted to Japan (Shimada et al. 2003).

Foliose *Ulva* species are morphologically very simple and show relatively few and subtle morphological differences (Flagella et al. 2010, Lee et al. 2019), with overlaps between species morphologies, which raises questions about their genetic distinctness. Indeed, with such small phenotypical distinctions, one might expect their genetic diversity to be limited. However, barcoding data might be insufficient to characterize the genetic diversity between species since the barcodes only represent a small proportion of the entire genetic information of an individual, and have been originally selected for species delimitation more than for intra‐specific variation (Kress and Erickson 2008). As such, larger scale sequencing is required to precisely measure the inter‐ (and intra‐) specific genetic diversity the foliose *Ulva* species found in our dataset. Hence, we sequenced and analyzed the organellar genomes of all of the 110 strains used in this study, which enabled us to characterize the extent of genetic variation between and within the 6 sampled *Ulva* species. Strikingly, we found i) extensive variation in the organellar genome sizes among the 6 species, and ii) high levels of single nucleotide polymorphisms (SNPs) between species. Organellar genomes sizes varied by 24 kb (94 to 118 kb) and 29 kb (59 to 88 kb) between species in their chloroplast and mitochondrion, respectively. These results are in line with previous reports for seven *Ulva* species chloroplasts: (*U*. *flexuosa,* Cai et al. 2017; *U. fasciata,* Melton and Lopez‐Bautista 2017; *U. ohnoi,* Suzuki et al. 2018; *U. mutabilis*, NCBI reference sequence NC_043860; *U. prolifera,* NCBI reference sequence NC_036137; *U. linza,* Wang et al. 2017; and *U. lactuca,* Hughey et al. 2019) as well as mitochondrion: (*U. linza,* Zhou et al. 2016; *U.fasciata,* Melton and Lopez‐Bautista 2016; *U. pertusa,* Liu et al. 2017; *U. expansa,* Hughey et al. 2018; *U. flexuosa,* Cai et al. 2018; *U. compressa,* NCBI reference sequence NC_043860; and *U. mutabilis*, NCBI accession MK069587). Such genome size variation is not however expected to underlie functional diversification of the organellar genomes as gene count estimates did not vary widely among the six species (100 to 102 and 57 to 61 for chloroplast and mitochondria, respectively; Tables 1 and 2). Hence, the size variation is likely explained by the presence or absence of introns or varying amounts of intergenic DNA, such as was recently reported in the commercial red alga *Pyropia yezoensis* (Xu et al. 2019). SNPs difference between species are abundant with ~ 5,000 SNPs between *U. laetevirens* and *U. australis* in both of their organellar coding sequences, indicating that despite being morphologically similar, extensive cytoplasmic genetic variation is present between *Ulva* species. For comparison, the chloroplast of kelp species display ~ 30 SNPs · kb^‐1^ (Rana et al. 2019), as opposed to as high as 82 SNPs kb‐1 in *Ulva*, indicating a large inter‐specific variation. Compared with land plants, *Ulva* inter‐specific variation in organellar DNA is significantly higher than that reported for ginseng species (~7 SNPs · kb^‐1^ between seven species; Giang et al. 2020), 1.4 SNPs · kb^‐1^ for six *Perilla* species (Cheon et al. 2018) or ~ 25 SNPs · kb^‐1^ between 25 oat (*Avena*) species (Fu 2018). Thus, our data clearly show that foliose *Ulva* species are indeed separate taxa, with clear separation between species and a previously unknown high inter‐specific organellar genetic diversity (Figs. 1 and 3).

Intra‐specific variation in the entire organelle sequences was relatively limited, with 0.03 to 1.98 SNPs · kb^‐1^ between individuals of the same species (Table 4) despite including non‐coding regions which are expected to evolve under different selective pressures (Kelchner 2000). For instance, the 29 *Ulva rigida* individuals in this study only carried 117 and 36 variant sites in their chloroplast and mitochondrion, respectively. Given the limited cytoplasmic genetic variation, we expect that the large growth and metabolic differences between *Ulva* individuals from the same species (Fort et al. 2019) is probably largely explained by variation in the nuclear genome. Our strategy did not allow for precise characterization of nuclear genome diversity due to limited coverage and high abundances of bacterial DNA. Such characterization of the nuclear genetic diversity of *Ulva* species and association of possible genetic markers with growth and nutritional variation will be an important part of future strain selection efforts. An analysis of the nuclear genome of the *Ulva* species presented here will require the generation of reference genome(s) for all six species. Indeed, the large genetic variation in the organellar genomes likely indicates a similar genetic variation in the nuclear genome, and SNP analysis should be performed using a reference for each individual species. Future efforts will be needed to generate axenic cultures of all six species, using methods described by (Califano et al. 2018, De Clerck et al. 2018), to avoid DNA contamination from *Ulva* symbionts (Alsufyani et al. 2020).

Structural rearrangements within the organellar genome of organisms of different species can provide valuable information regarding the evolutionary history of the lineages (Downie and Palmer 1992, Gao et al. 2009, Ng et al. 2017). Here, we found a large structural rearrangement between the six *Ulva* species and characterized as an inversion of a large portion of the chloroplast DNA between a clade comprised of *U. rigida*/ *U. laetevirens* and/ *U. gigantea,* and another that included *U. fenestrata*/ *U. australis* and/ *U. pseudorotundata* (Fig. 2). The orientation of this region in the other published plastid genomes of *Ulva* (Fig. S6; *U. mutabilis*, *U. fasciata*, *U. lactuca*, *U. ohnoi*, *U. flexuosa*, *U. linza,* and *U. prolifera*), was conserved with the former clade. Thus, we expect *U. fenestrata*, *U. australis,* and *U. pseudorotundata* to separate from the other *Ulva* taxa through a shared ancestral speciation event.

### Traditional barcoding is appropriate for species classification in foliose *Ulva* spp

Foliose *Ulva* taxonomy has a long history of misidentifications and cryptic species (Wolf et al. 2012, Hughey et al. 2019, Steinhagen et al. 2019b). Interestingly, no cryptic species were detected among our sample set, indicating that the molecular investigations in the genus *Ulva* done worldwide cover a large part of the specific diversity of foliose *Ulva* in NE Atlantic and that the major challenge is to solve misidentification issues in the literature and databases.

Additionally, the use of a single, two, even three barcodes (Du et al. 2014, Chávez‐Sánchez et al. 2019) to classify the species of an individual deserves examination. We compare here the phylogenetic trees obtained from each of two chloroplast barcodes (*rbc*L and *tuf*A), and those obtained using the entire coding sequence of the chloroplast and mitochondrial genomes.

When comparing the Maximum Likelihood trees obtained with barcodes or the organellar coding sequence, we did not find any large difference between these tree structures or resulting clustering (Fig. 3). Each individual strain was attributed to the same taxa cluster using barcodes or the full coding sequences. The only difference found here was the bootstrapping and/or Bayesian posterior probabilities support for the tree nodes, with higher support when the entire coding sequence of the organelles was considered. Interestingly, while the clustering was similar, the bootstrap support was higher in *tuf*A than in *rbc*L for all species‐specific nodes. This result shows that a single barcode such as *tuf*A is sufficient for robust classification of foliose *Ulva* species in the NE Atlantic, in agreement with a previous report (Saunders and Kucera 2010), and this is likely to be the case elsewhere in the world. While Saunders and Kucera (2010) showed moderate success with *rbc*L as universal barcode for green macroalgae, we show here that *rbc*L is adequate when (i) the entire coding sequence is considered and (ii) the analysis is restricted to foliose *Ulva* species. As stated above however, only a handful of SNPs are found in the cytoplasmic genomes within each species. As such, the variation captured in the larger genomic region adds very little to the species tree construction other than higher confidence estimates to support the tree nodes (Fig. 3). One application where this variation can however contribute is in the determination of geographic origin, but our sampling frequency for each species at different location is likely too low to produce robust clustering.

### Inter‐species hybridization is a rare occurrence among *Ulva* species

The question arises as to what the capability of individuals from different *Ulva* species to hybridize in the wild is. In addition to the matter of species concepts, and defining species boundaries (Hiraoka et al. 2017), such hybrids could have considerable implications for *Ulva* strain selection and population genetics. Despite their established utility in characterizing evolutionary history, cytoplasmic markers cannot on their own indicate whether *Ulva* species are capable of hybridization due to a uniparental pattern of cytoplasmic inheritance as is seen in angiosperms (Hagemann 2004). Indeed, the chloroplast (or chloroplast DNA) of one parent is eliminated shortly after the fertilization of *Ulva* gametes (Bråten 1973, Kagami et al. 2008). In our study, we extracted the 45S rRNA sequence of the nuclear genome for each *Ulva* individual and constructed a phylogenetic tree. As with trees constructed using cytoplasmic genomes, *Ulva* species were readily distinguished, with as high as 65 high‐quality SNPs between *U. laetevirens* and *U. pseudorotundata* in the ~ 5 kb region corresponding to the 45S rRNA. The tree topology was also strikingly similar, with all but one individual being assigned the same species as with the cytoplasmic DNA. The exception (U99, originating from Portugal) was assigned as *U. rigida* using the 45S sequence and *U. laetevirens* using the cytoplasmic genomes (Fig. 4). Analysis of allelic frequencies revealed that approximately half of this strains’ 45S sequences possess *U. rigida* variants, and the other half were variants attributed to *U. laetevirens* (Fig. 4, B and C; Table S3 in the Supporting Information). Such a pattern can only reasonably be explained by this individual representing an F1 hybrid between the two species.

Our F1 hybrid was notably homoplasmic for both cytoplasmic genomes, validating the previous reports of uniparental inheritance of chloroplast DNA (Bråten 1973, Kagami et al. 2008). While those previous reports demonstrated uniparental inheritance of *Ulva compressa* and *U. mutabilis* chloroplast, both *Ulva* species that are likely conspecific (Steinhagen et al. 2019a), Figure 3, we extend those findings further with the same observation between two foliose *Ulva* species. Additionally, the mitochondrial homoplasmy in the hybrid U99 demonstrates the uniparental inheritance of the mitochondrial DNA. The mechanistic basis for such inheritance requires further study and induced hybridity between *U. rigida* and *U. laetevirens* could be a useful tool to shed light on this phenomenon. Notably, whether uniparental inheritance remains the rule when more hybrids are considered, including between other species, remains to be investigated.

The single occurrence of an inter‐species hybrid in> 100 foliose *Ulva* strains from six different species indicates that the foliose *Ulva* species investigated here are generally unable to produce viable hybrid offspring in the wild, with the rare exception of one example between *U. rigida* and *U. laetevirens.* Of note, *U. rigida* and *U. laetevirens* are closely related species, and potential natural hybridization events between more distantly related or between tubular and foliose species remain to be thoroughly investigated. Also, the presence of F2 and later hybrids cannot fully be investigated in this system, since additional nuclear markers need to be assessed to investigate more “ancestral” hybridization events. However, the lack of discrepancy between the single nuclear marker and the organelles indicates generally rare, if any, hybridization events between foliose species. The fact that inter‐species hybrids between *U. rigida* and *U. laetevirens* are possible but that their presence is rare likely indicates that a postzygotic barrier (such as low hybrid fitness, Arnold et al. 2012; and zygote lethality, Bushell et al. 2003, Fort et al. 2017) prevents such hybrids to spread through the population. The life history of *Ulva*, with its facultative sexual reproduction, ability to propagate vegetatively and sometimes clonal apomictic reproduction (Ogawa et al. 2015) likely also plays a role in such reproductive isolation. Finally, since *Ulva* species have been shown to be either anisogamous or isogamous (Smith 1947), pre‐fertilization barriers to inter‐species hybridization are also likely present. More extensive sampling (i.e., more individuals analyzed in zones rich in species diversity) might reveal a greater extent of hybridization between *Ulva* species and will be the focus of future studies. Interestingly, studies involving the ITS barcode (part of the 45S rRNA gene) have previously been reported to show some discrepancy with the results of a cytoplasmic barcodes (*rbc*L or *tuf*A; Lawton et al. 2013). Those discrepancies were also suggested to be attributable to the presence of a hybrid among the sampled individuals; however, no further validation of this suggestion was conducted in this instance. Revisiting Sanger sequencing data from these experiments with consideration for estimates of allelic frequency, although less robust than that achieved through library sequencing, may be useful in identifying other examples of wild hybridization between foliose *Ulva* species. Library sequencing gives reliable allele frequencies estimates across the entire 45S repeats, and is, unlike Sanger sequencing, not sensitive to the presence of amplicons of different sequences and/or lengths within a PCR product.

An examination of the possibility for creation and use of foliose *Ulva* hybrids in aquaculture represents an exciting venture for future studies. Such approaches are responsible for significant improvements, termed hybrid vigor or heterosis, in desirable characteristics as compared to parental lines in land plants (McKeown et al. 2013, Fort et al. 2016). Does inter‐species hybrid vigor occur in *Ulva*? Previous reports indicated that tubular sister species *U. prolifera* and *U. linza* can hybridize (Hiraoka et al. 2011), but this was not reported to be associated with any hybrid vigor (Xie et al. 2020). On the other hand, protoplast fusion between *Monostroma oxyspermum* and *Ulva reticulata* were viable and some hybrids showed evidence of heterosis (Gupta et al. 2015). Whether this outcome will hold true in foliose *Ulva* from relatively divergent species remains to be investigated. Another common feature of inter‐species hybrids is a high frequency of infertility, and *Ulva* hybrids may similarly show reduced capacity of fertility and/or sporulation, a property that may be rendering them extremely attractive in an industrial system.

This work was funded by the European Union Horizon 2020 programme (project ID 727892, GenialG ‐ GENetic diversity exploitation for Innovative Macro‐ALGal biorefinery, http://genialgproject.eu/) and Science Foundation Ireland Frontiers for the Future (Project Pristine Coast, award no 19/FFP/6841). The authors would like to thank Isabel Azevedo (CIIMAR), Ricardo Bermejo (NUI Galway), Adrie van der Werf (Wageningen University), Paolo Ruggeri (Station Biologique de Roscoff) and Helena Abreu (Alga +) for providing some of the strains used in this study. Björn Usadel acknowledges the Ministry of Innovation, Science, and Research within the framework of the NRW Strategieprojekt BioSC (No. 613 313/323‐400‐002 13) and the Excellence Cluster CEPLAS (EXC 1028). The authors have no conflict of interest to declare.

## Supporting information


**Figure S1.** Log likelihood of the Bayesian Poisson Tree Process MCMC iterations.

**Figure S2.** Principal Component Analysis of SNPs of the 110 strains mapped against the chloroplast assembly of strain U41. PC1 to PC5 are shown.

**Figure S3.** Flowchart of the de novo organelle assembly.

**Figure S4.** Chloroplast annotation of each of the six species.

**Figure S5.** Mitochondrion annotation of each of the six species.

**Figure S6.** Mauve alignment of the chloroplast genome of the six species in this study, as well as seven previously published Ulva species.

**Figure S7.** Mauve alignment of the mitochondrial genome of the six species in this study, as well as eight previously published Ulva species.

**Figure S8.** Principal Component Analysis of SNPs of the 110 strains mapped against U.laetevirens strain U41 nuclear 45S ribosomal RNA repeats. PC1 to PC5 are shown.

**Table S1.** Name, location, species, and NCBI reference numbers for each strain in this study.

**Table S2.** Common set of protein‐coding genes used for organelle coding sequence analysis.

**Table S3.** 45S rRNA repeats allelic ratios of hybrid strain U99.
