# Supplementary figures and images for: Foliose Ulva Species Show Considerable Inter‐Specific Genetic Diversity, Low Intra‐Specific Genetic Variation, and the Rare Occurrence of Inter‐Specific Hybrids in the Wild
Source: J Phycol. 2020 Nov 24;57(1):219–33. doi: 10.1111/jpy.13079 (PMC7894351; doi:10.1111/jpy.13079)

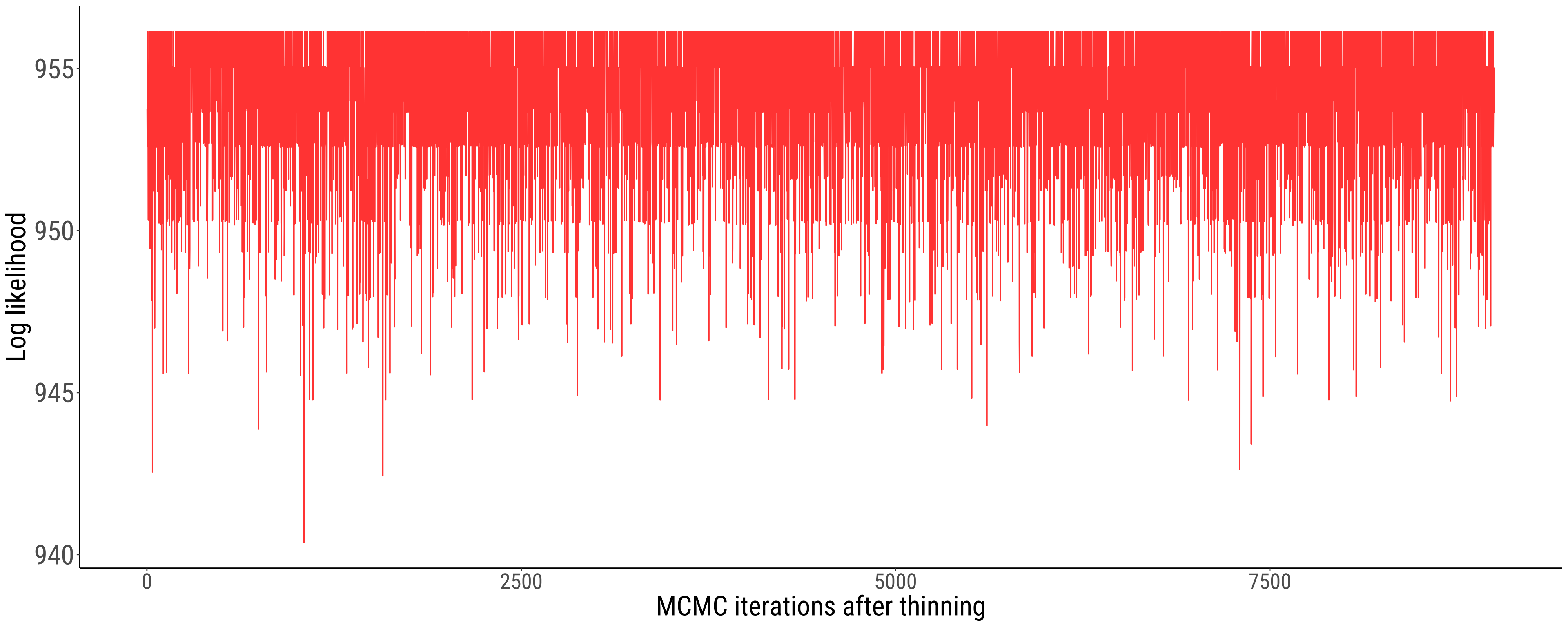

Supplement: Supplementary file 1 — Figure S1. Log likelihood of the Bayesian Poisson Tree Process MCMC iterations. [file JPY-57-219-s001.pdf]

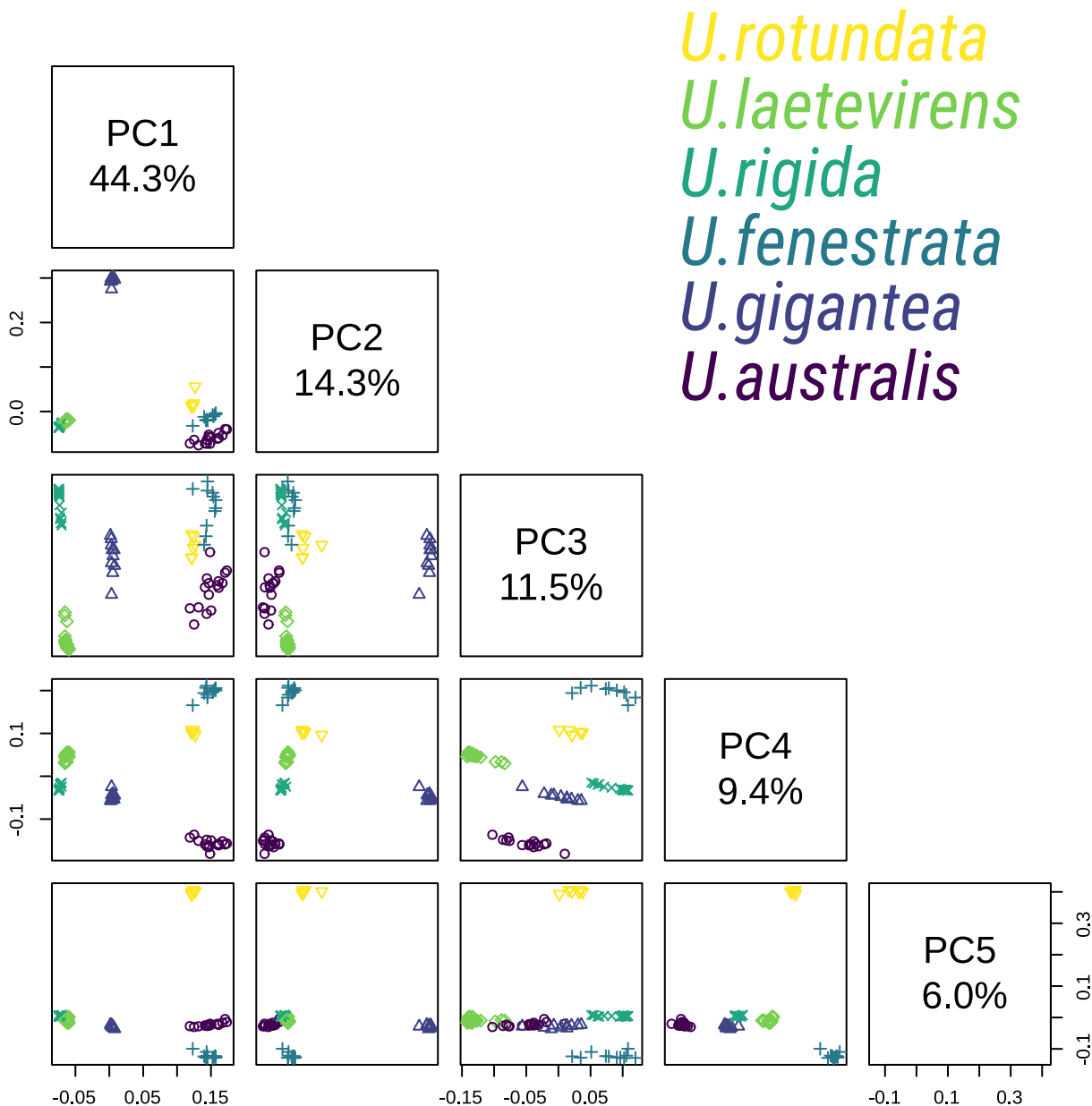

Supplement: Supplementary file 2 — Figure S2. Principal Component Analysis of SNPs of the 110 strains mapped against the chloroplast assembly of strain U41. PC1 to PC5 are shown. [file JPY-57-219-s002.pdf]

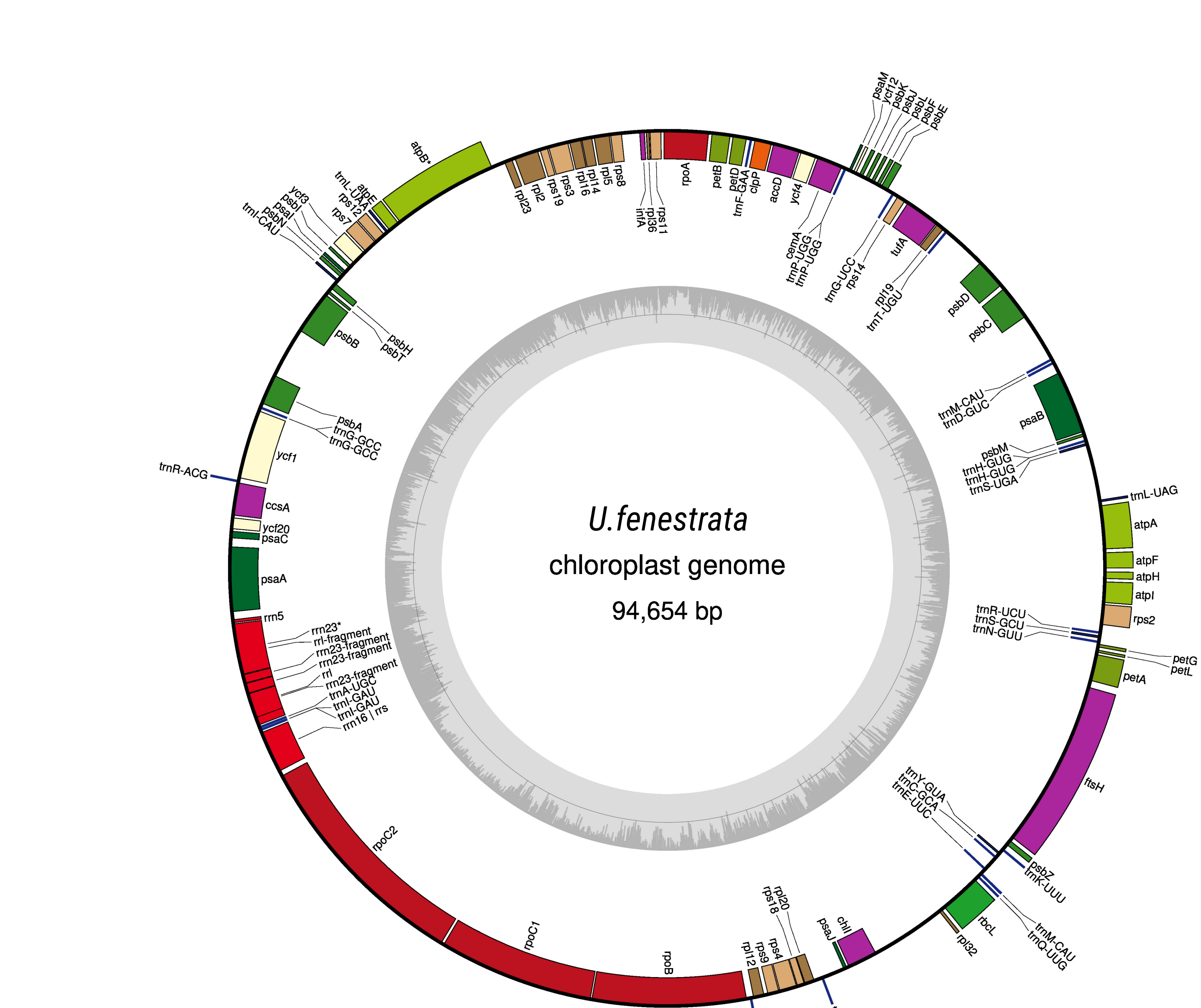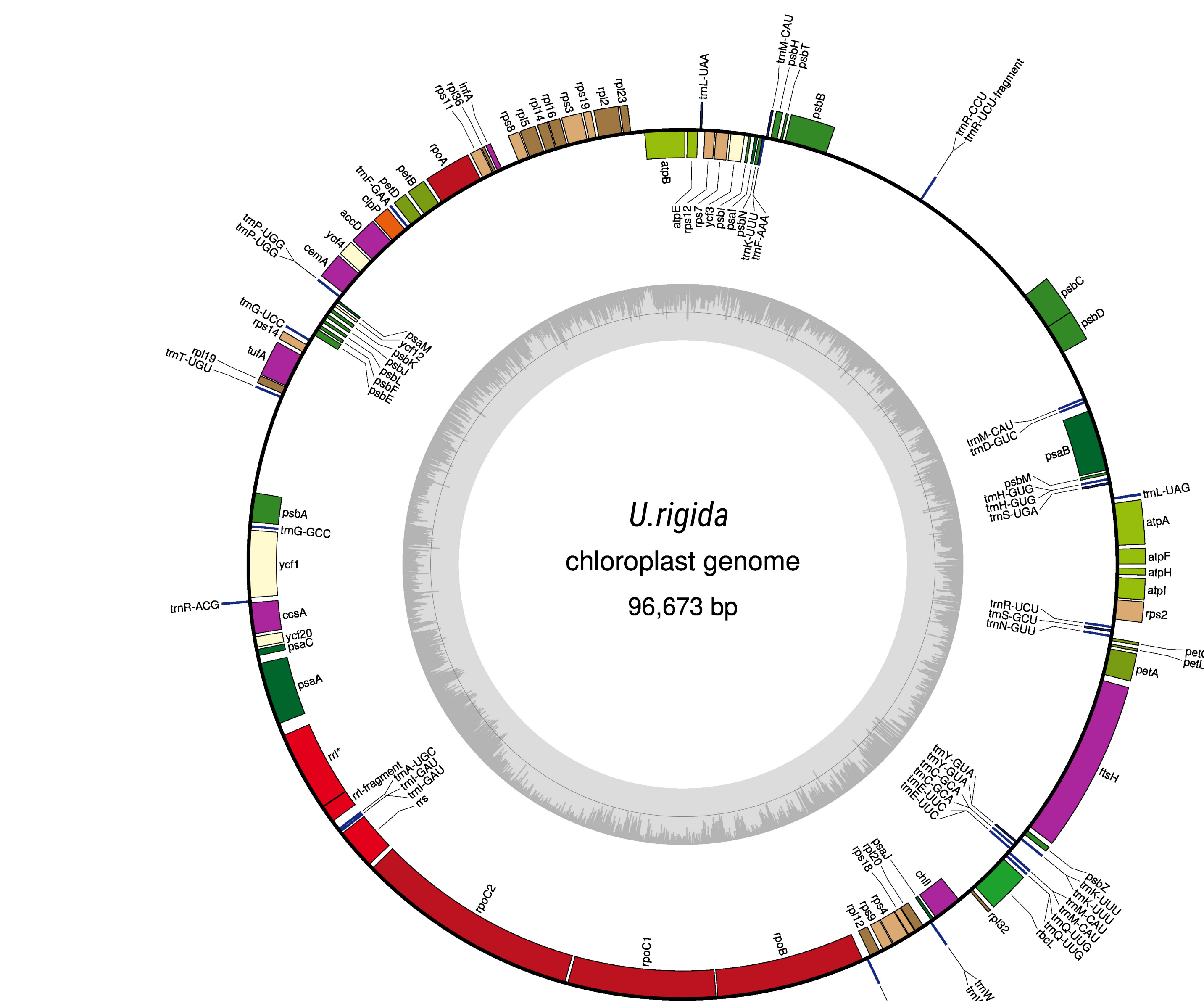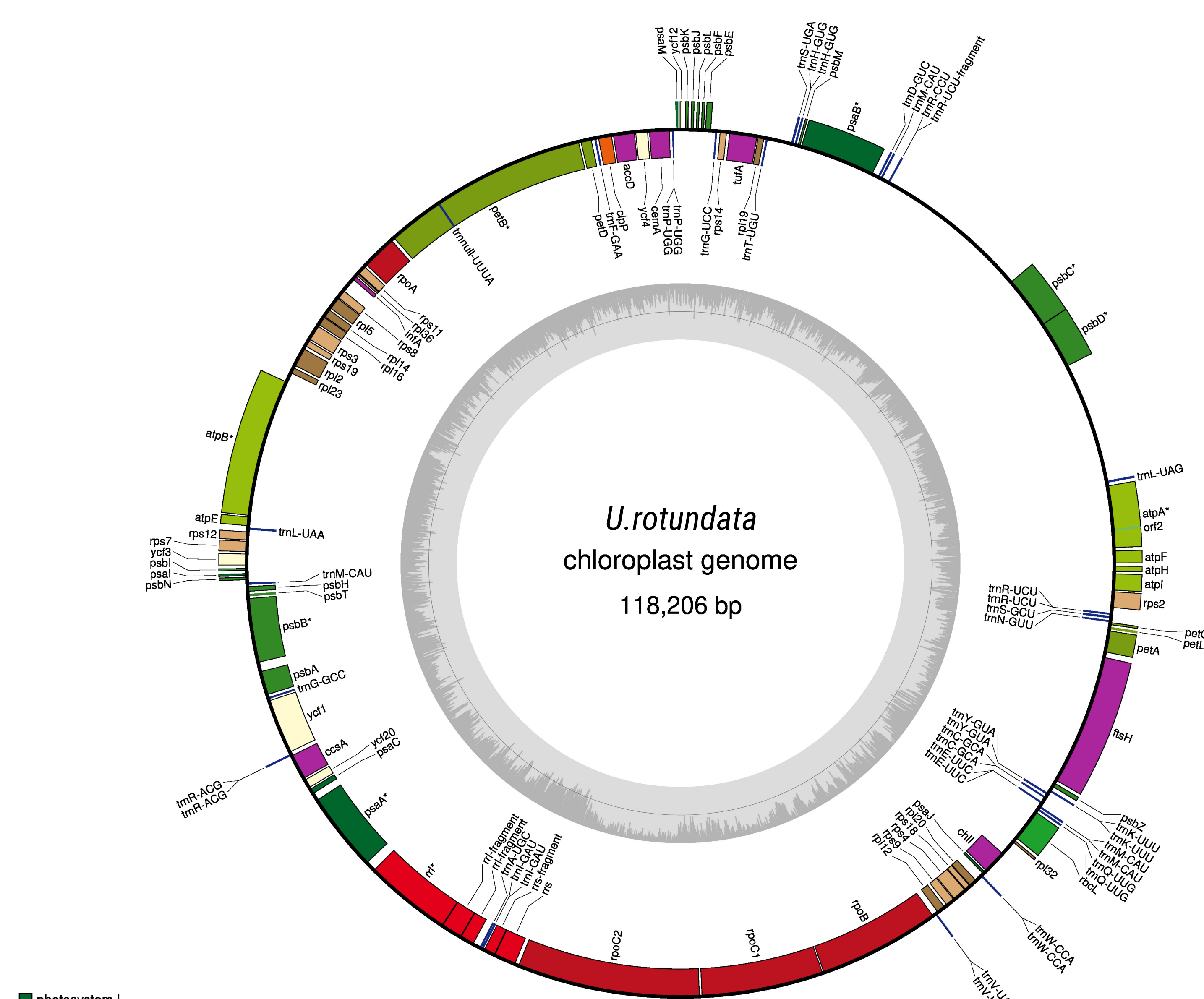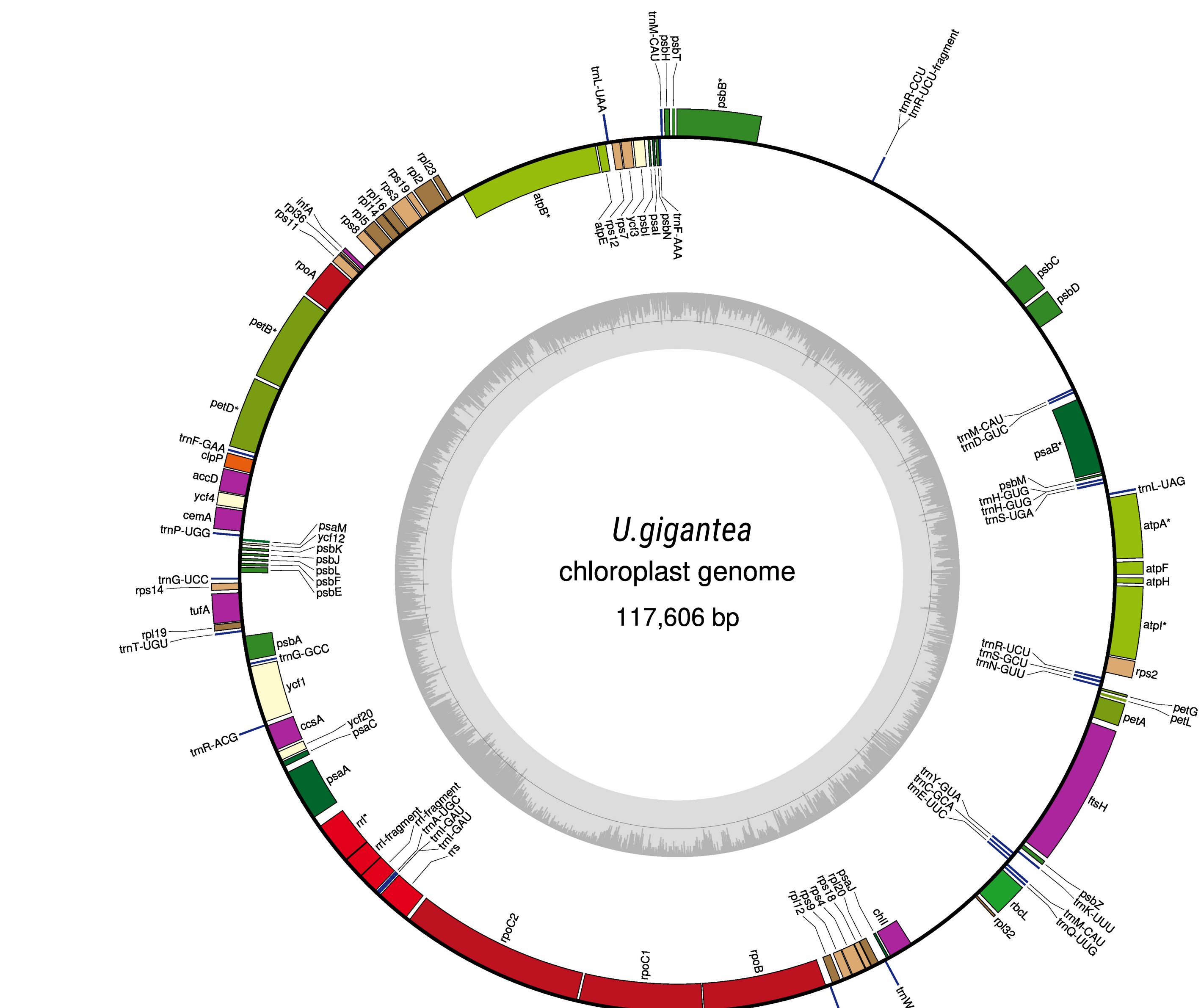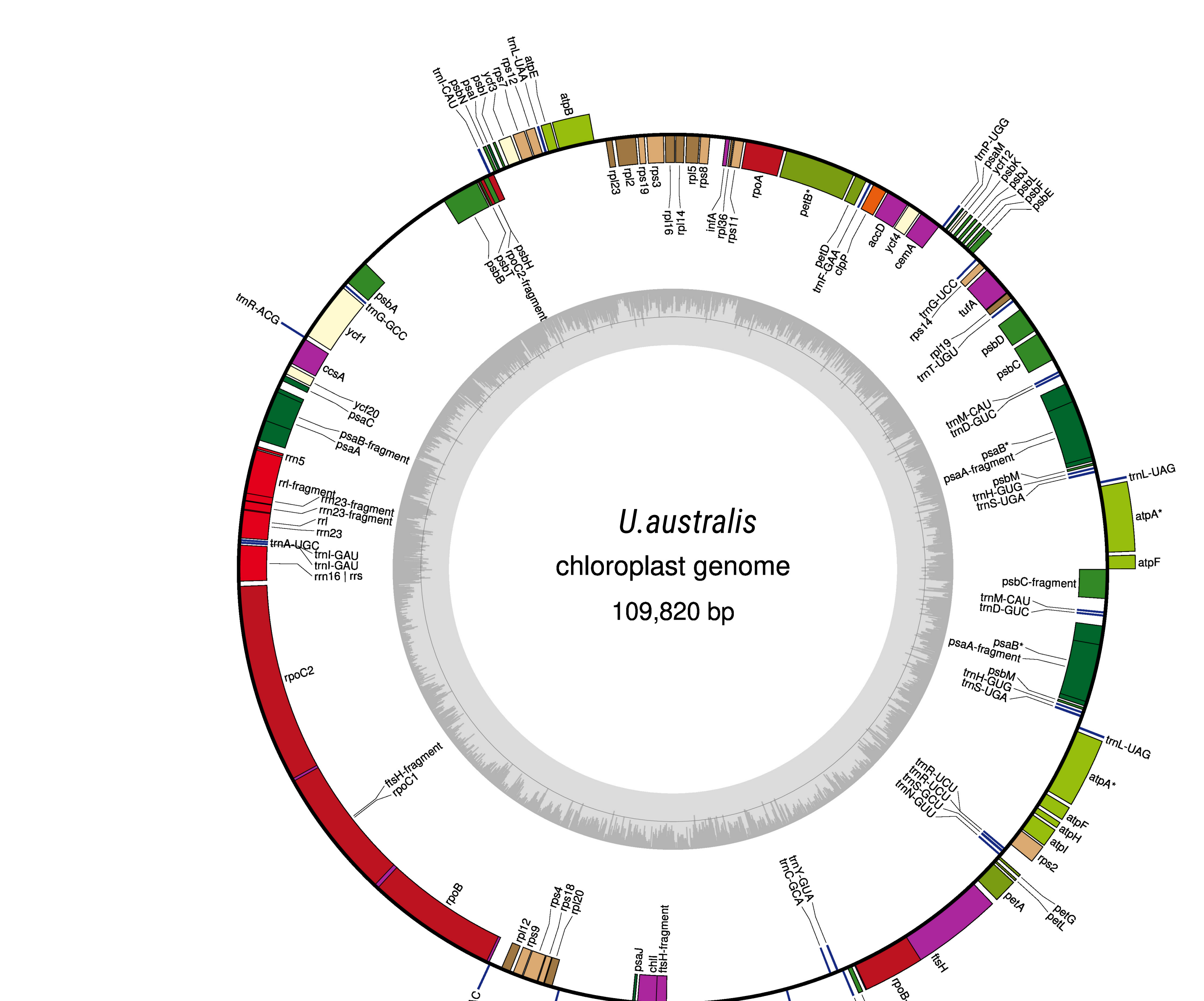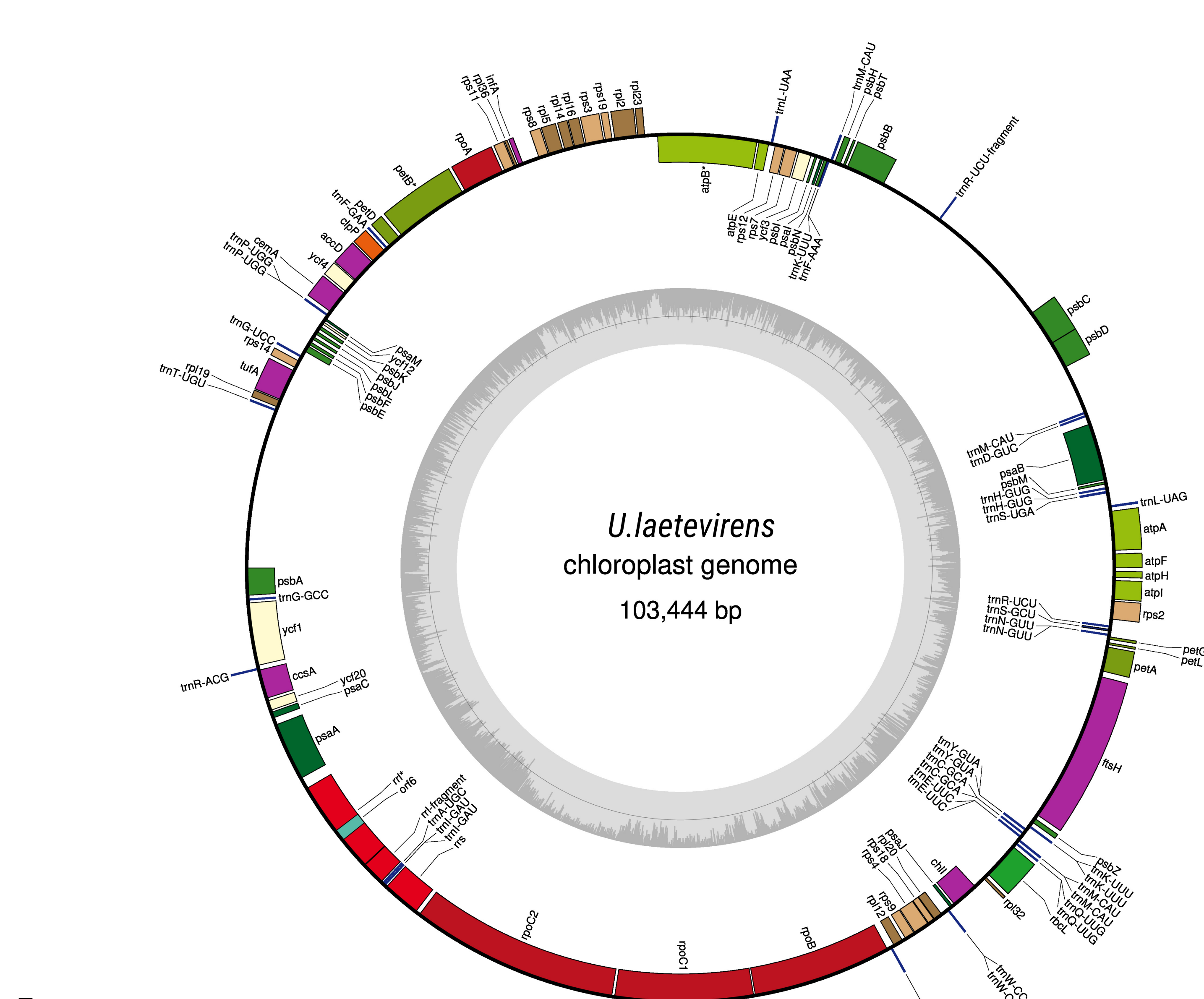

Supplement: Supplementary file 4 — Figure S4. Chloroplast annotation of each of the six species. [file JPY-57-219-s004.pdf]

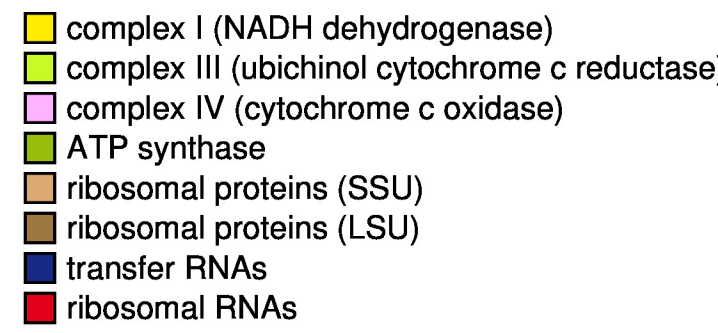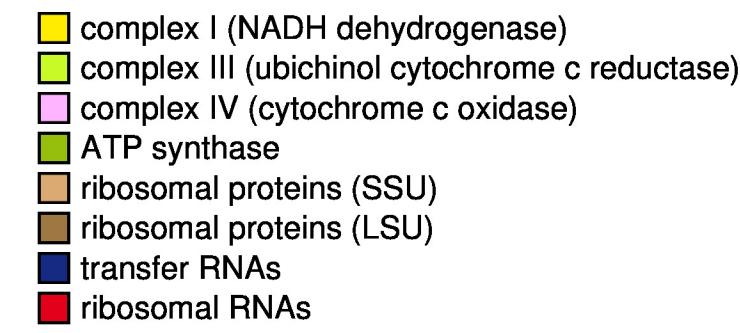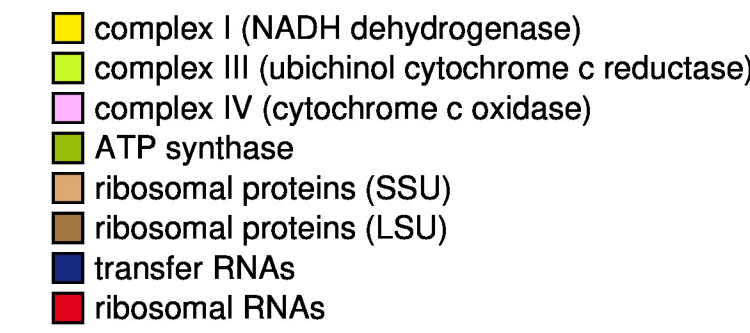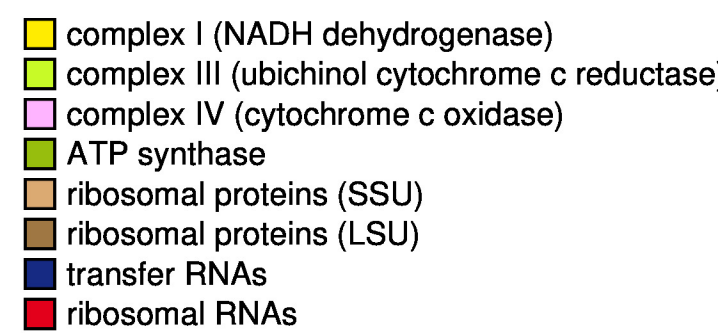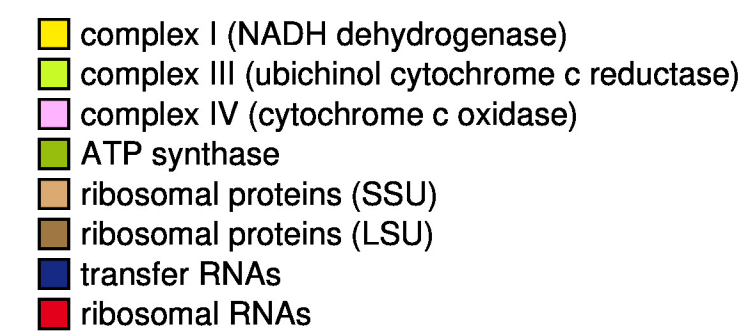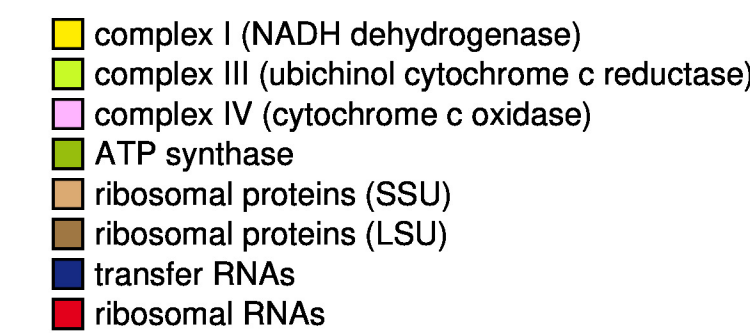

Supplement: Supplementary file 5 — Figure S5. Mitochondrion annotation of each of the six species. [file JPY-57-219-s005.pdf]

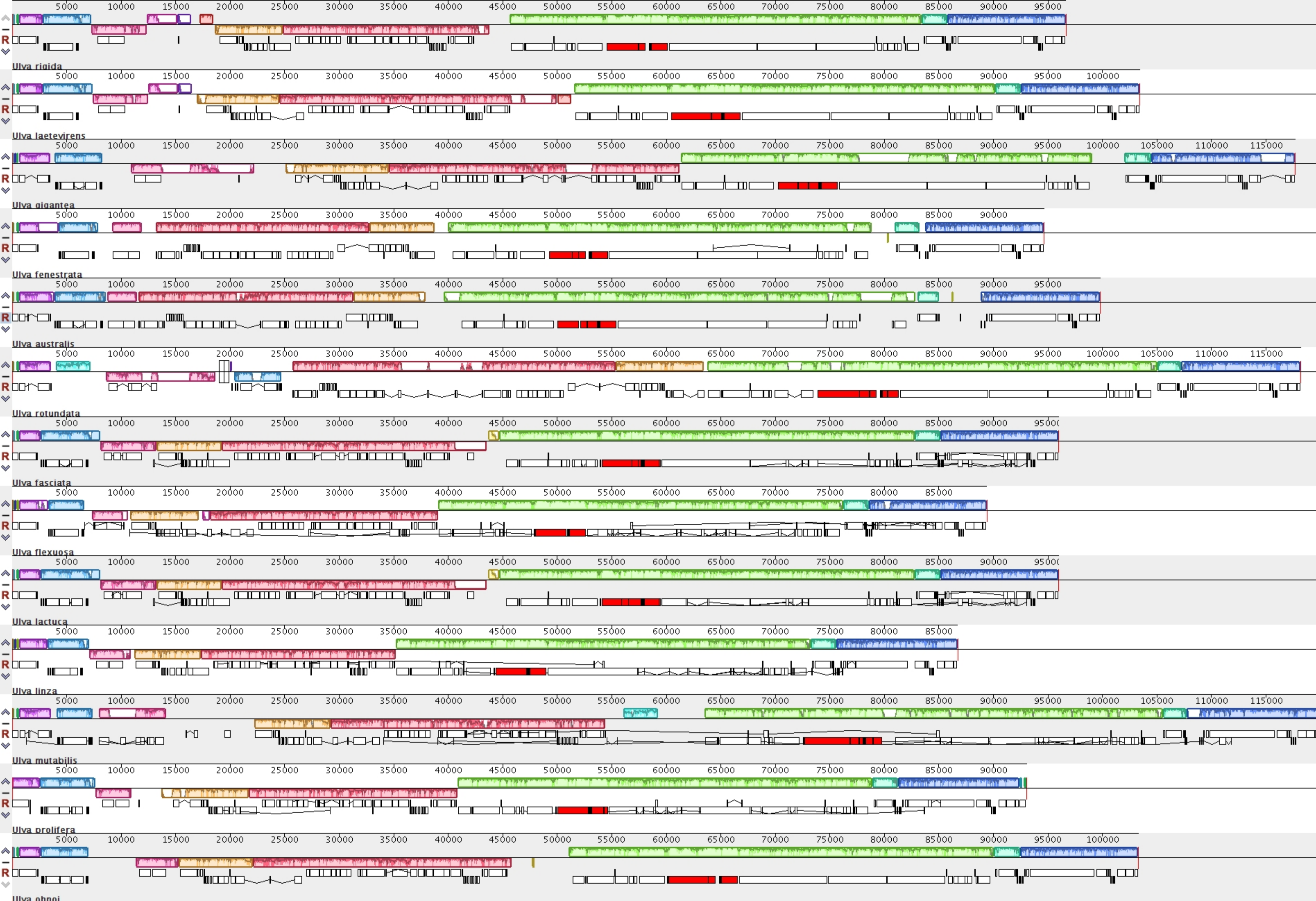

Supplement: Supplementary file 6 — Figure S6. Mauve alignment of the chloroplast genome of the six species in this study, as well as seven previously published Ulva species. [file JPY-57-219-s006.pdf]

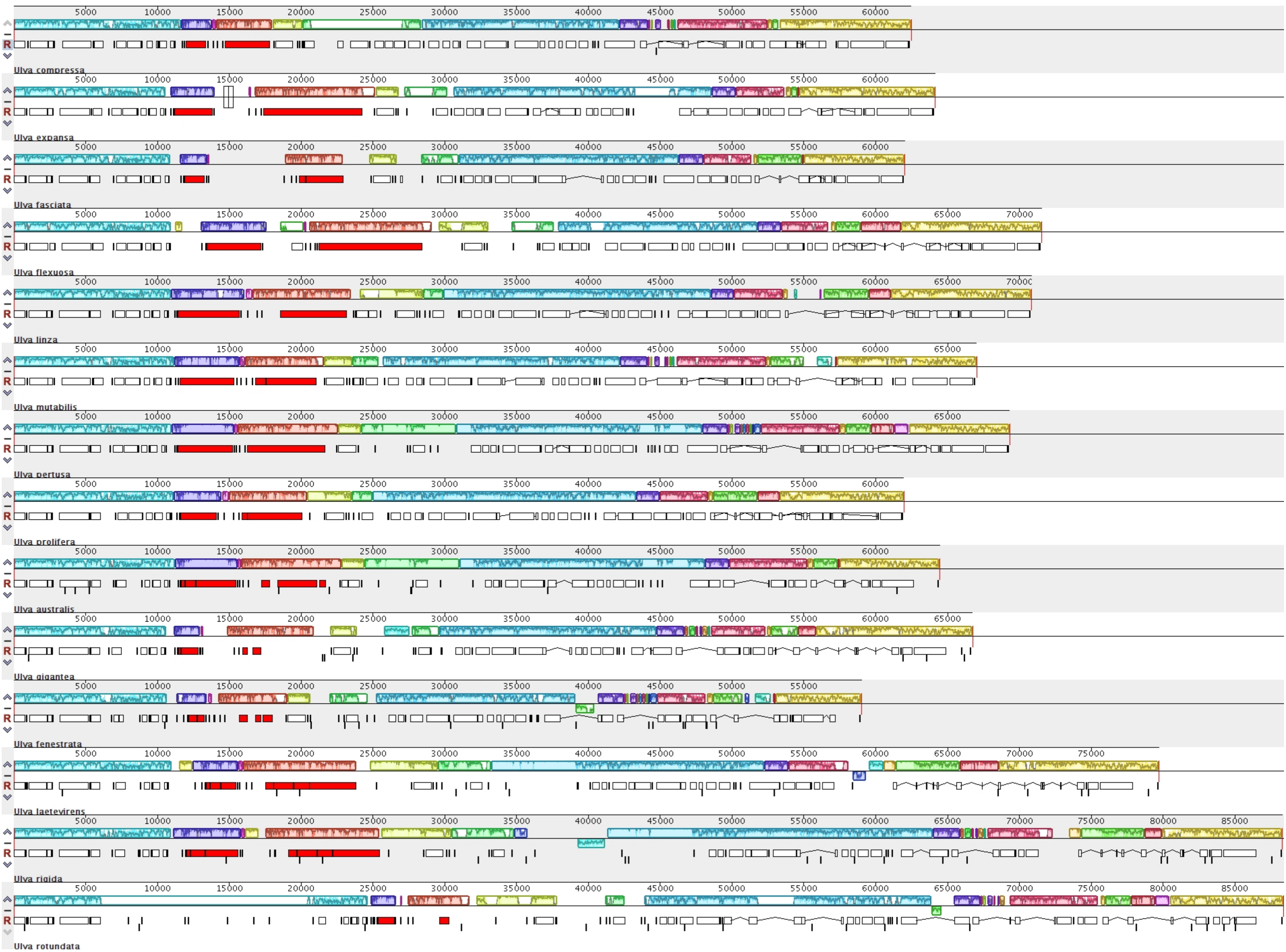

Supplement: Supplementary file 7 — Figure S7. Mauve alignment of the mitochondrial genome of the six species in this study, as well as eight previously published Ulva species. [file JPY-57-219-s007.pdf]

*U. rotundata*  
*U. rigida*  
*U. laetevirens*  
*U. fenestrata*  
*U. gigantea*  
*U. australis*

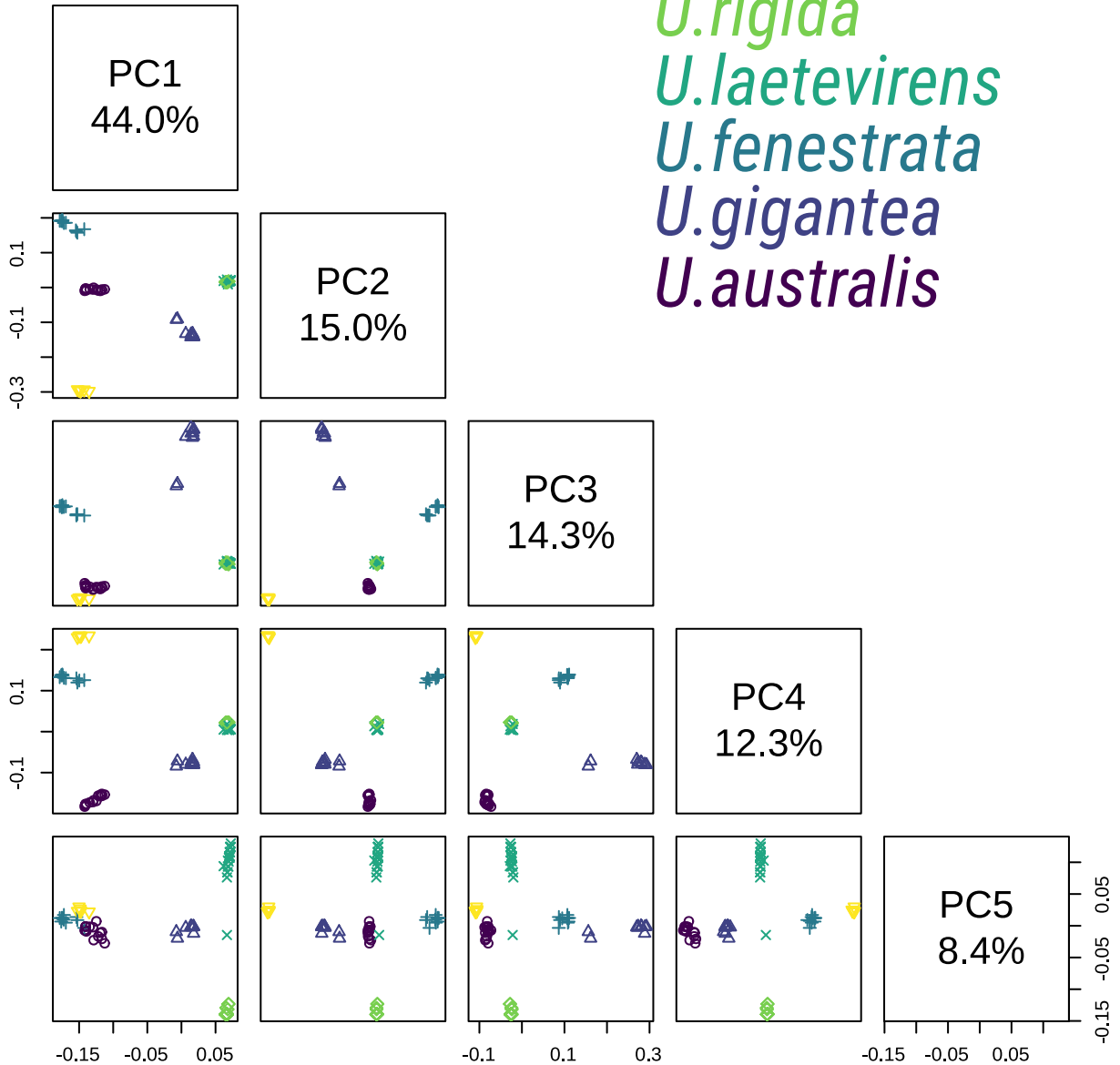

Supplement: Supplementary file 8 — Figure S8. Principal Component Analysis of SNPs of the 110 strains mapped against U.laetevirens strain U41 nuclear 45S ribosomal RNA repeats. PC1 to PC5 are shown. [file JPY-57-219-s008.pdf]
