# Supplementary material for: Foliose Ulva Species Show Considerable Inter‐Specific Genetic Diversity, Low Intra‐Specific Genetic Variation, and the Rare Occurrence of Inter‐Specific Hybrids in the Wild
Source: J Phycol. 2020 Nov 24;57(1):219–33. doi: 10.1111/jpy.13079 (PMC7894351; doi:10.1111/jpy.13079)
Supplement: Supplementary file 3 — Figure S3. Flowchart of the de novo organelle assembly. [file JPY-57-219-s003.pdf]

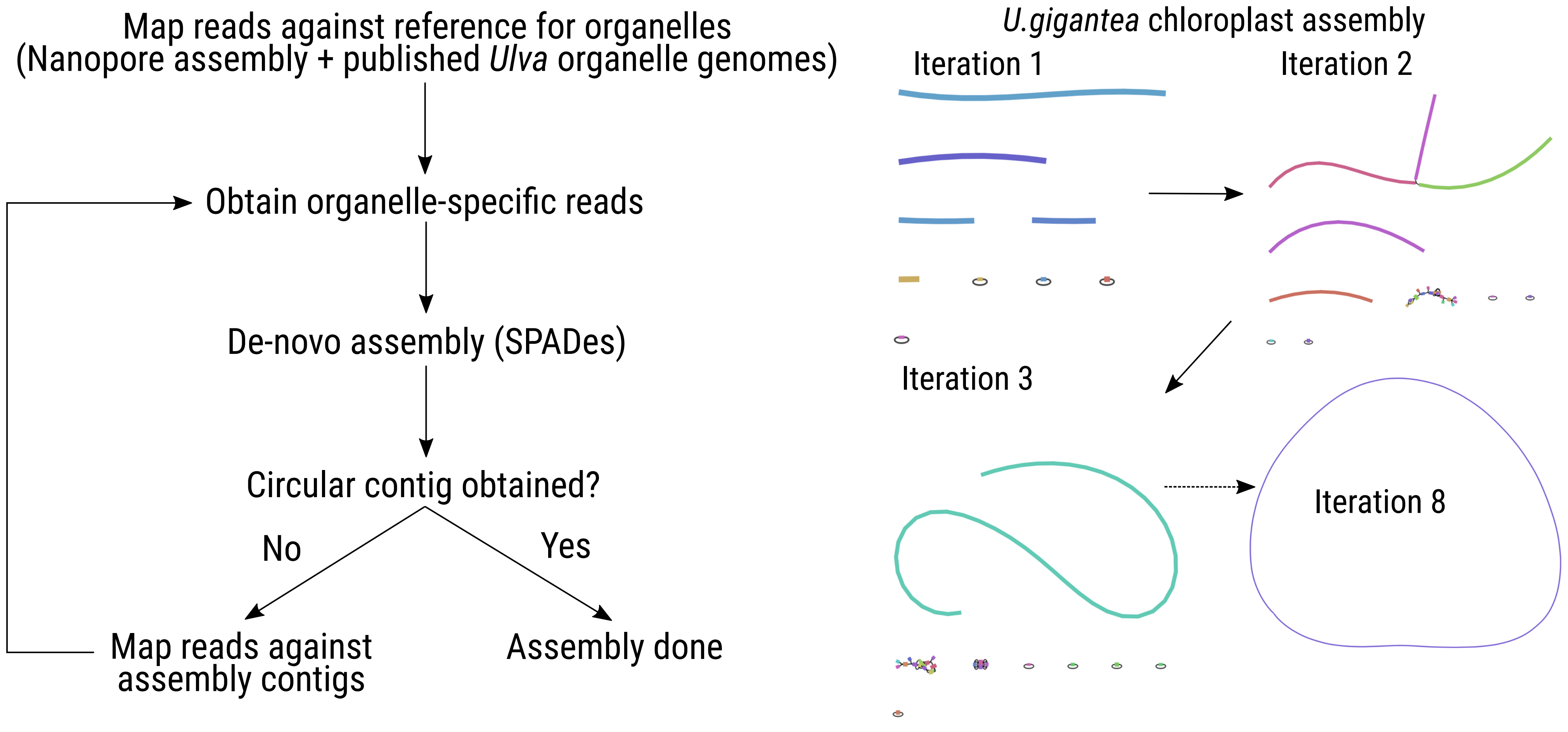

Map reads against reference for organelles
(Nanopore assembly + published *Ulva* organelle genomes)
Obtain organelle-specific reads
De-novo assembly (SPADes)
Circular contig obtained?
No
Yes
Map reads against assembly contigs
Assembly done
U.gigantea chloroplast assembly
Iteration 1
Iteration 2
Iteration 3
Iteration 8
